# The Value of Wetlands in Protecting Southeast Louisiana from Hurricane Storm Surges

**DOI:** 10.1371/journal.pone.0058715

**Published:** 2013-03-11

**Authors:** Edward B. Barbier, Ioannis Y. Georgiou, Brian Enchelmeyer, Denise J. Reed

**Affiliations:** 1 Department of Economics and Finance, University of Wyoming, Laramie, Wyoming, United States of America; 2 Department of Earth and Environmental Sciences and Pontchartrain Institute for Environmental Sciences, University of New Orleans, New Orleans, Louisiana, United States of America; The Ohio State University, United States of America

## Abstract

The Indian Ocean tsunami in 2004 and Hurricanes Katrina and Rita in 2005 have spurred global interest in the role of coastal wetlands and vegetation in reducing storm surge and flood damages. Evidence that coastal wetlands reduce storm surge and attenuate waves is often cited in support of restoring Gulf Coast wetlands to protect coastal communities and property from hurricane damage. Yet interdisciplinary studies combining hydrodynamic and economic analysis to explore this relationship for temperate marshes in the Gulf are lacking. By combining hydrodynamic analysis of simulated hurricane storm surges and economic valuation of expected property damages, we show that the presence of coastal marshes and their vegetation has a demonstrable effect on reducing storm surge levels, thus generating significant values in terms of protecting property in southeast Louisiana. Simulations for four storms along a sea to land transect show that surge levels decline with wetland continuity and vegetation roughness. Regressions confirm that wetland continuity and vegetation along the transect are effective in reducing storm surge levels. A 0.1 increase in wetland continuity per meter reduces property damages for the average affected area analyzed in southeast Louisiana, which includes New Orleans, by $99-$133, and a 0.001 increase in vegetation roughness decreases damages by $24-$43. These reduced damages are equivalent to saving 3 to 5 and 1 to 2 properties per storm for the average area, respectively.

## Introduction

Field studies indicate that coastal marsh vegetation significantly impacts wave attenuation, as measured by reductions in wave height per unit distance across a wetland [1]–[3]. Such evidence is often cited to support marsh restoration globally for the purpose of protecting low-lying coastal communities and property from hurricanes and storms [4]–[10]. For example, global assessments of coastal wetland loss in temperate zones urge marsh restoration as a priority in protecting coastlines [8]–[10]. In Europe, the building of coastal defenses has accelerated marsh loss, thus increasing the vulnerability of coastal populations and property to storms [9]. Plans for wetland restoration along the US Gulf Coast have stepped up in the aftermath of the 2005 Hurricanes Katrina and Rita [4]–[7]. For example, the President’s Gulf Coast Ecosystem Restoration Task Force recommends extensive wetland restoration, given that the “Gulf’s wetlands provide a natural flood attenuation function, which may reduce the impacts of flooding associated with storms” [6]. Because of this growing global interest in wetland restoration to protect temperate coastlines, and the considerable cost involved in such restoration efforts, there is also a need for more studies on the economic benefits in terms of reducing storm damages [1]–[3]. Although there are an increasing number of studies of the role of tropical coastal wetlands in reducing casualties and damages from storm surges [11]–[15], there have been few economic valuations of the storm protection service of coastlines dominated by temperate marshes [16], [17].

To determine the value of the storm protection service of wetlands requires consideration of the varying hydrodynamic properties of storm surges as well as the effects of differing wetland landscape and vegetation conditions across coastal systems. Although previous studies for temperate coastal wetlands have lacked such data [16], [17], recent storm surge models developed for southern Louisiana show how the attenuation of surge by wetlands is affected by the bottom friction caused by vegetation, the surrounding coastal landscape, and the strength and duration of the storm forcing [18]–[21]. We show how the hydrodynamic outputs from these models can be used to estimate the storm protection benefits of wetlands to southeastern Louisiana, which includes greater New Orleans. Once the various influences of wetland landscape and vegetation on storm surge are determined, they can be applied to estimate the effects of wetlands on damage from flooding, based on standard modeling approaches that relate property damages to the flood depth caused by surges [22]–[27]. As damage estimates for Hurricane Katrina and other storms indicate, the most important flooding impact caused by hurricane storm surges along many temperate coastlines is to residential property [23], [26], [28], [30], [31]. The results of our analysis show that wetland continuity and vegetation roughness measured along a coastal transect are effective in reducing hurricane storm surge levels and thus demonstrate how wetland conditions can cause a significant reduction in property damage.

## Materials and Methods

We analyze the results of hurricane storm surge simulations and combine them with economic analysis of the expected damage to residential property damage. We use this combined analysis to determine the value of both the presence of marsh and the friction effect of its vegetation in terms of reducing storm surge damage.

We performed a storm surge transect analysis along a selected location in the Caernarvon Basin in southern Louisiana east of New Orleans (**Figure S1**). The transect orientation and location were determined by using pre-extracted hydrodynamic (surge) data from a previous study derived from validated numerical models [21], [22]. These models involve a basin to channel scale implementation of the Advanced Circulation (ADCIRC) unstructured grid hydrodynamic model, which has been developed to simulate hurricane storm surge, tides, and river flow in the Gulf Coast region in support of flood protection and restoration. The time-dependent surge elevations for the entire duration of each modeled storm (entire storm hydrograph including rise and fall to normal levels) were used to define the maximum storm surge elevation gradients along the transect.

**Figure 1:**
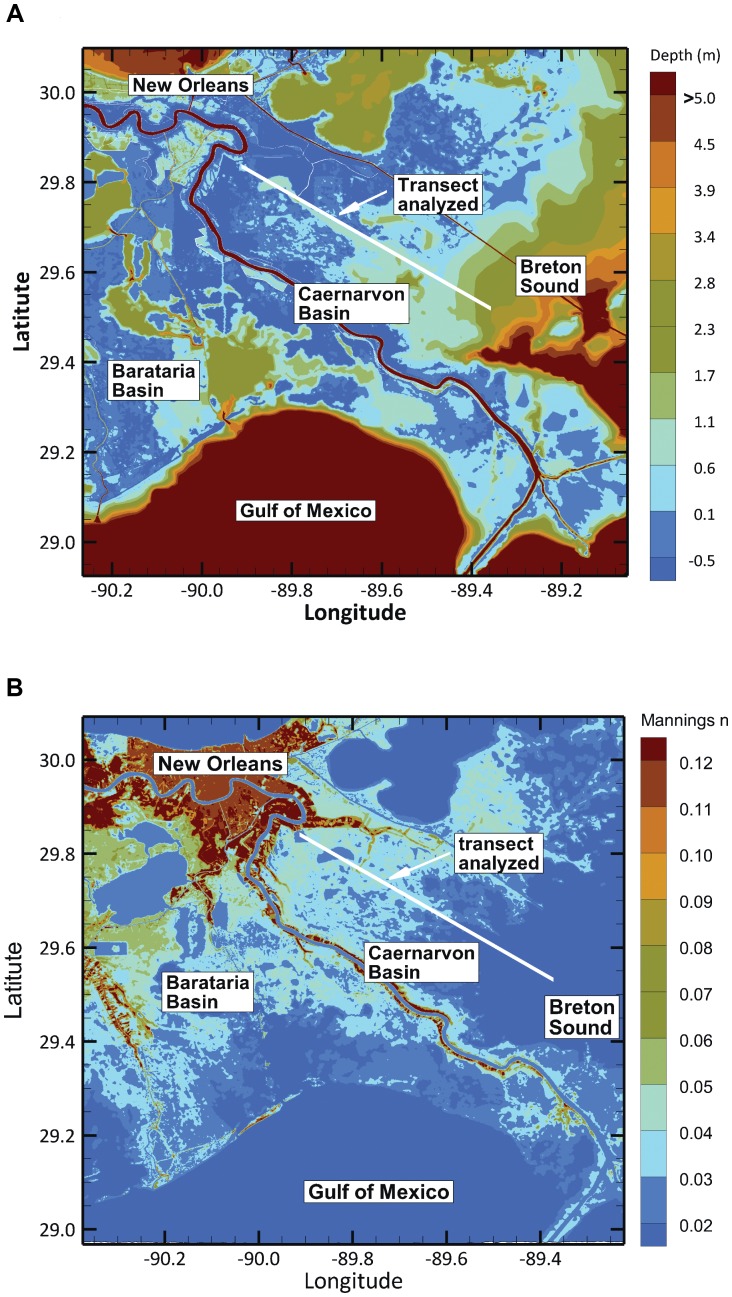
Location of the transect analysis for the Caernarvon Basin in southeast Louisiana. Panel A indicates the topo-bathymetric view used to obtain wetland-water ratio (W_L_) during the transect analysis. The color map shows depth below sea-level in meters, and the solid white line indicates the location of the transect where the analysis was performed. **Panel B** indicates habitat distribution used to obtain wetland roughness (W_R_) during the transect analysis. The color map shows the value for the Manning’s n indicator of bottom friction, and the solid white line indicates the location of the transect where the analysis was performed.

Along the transect for the Caernarvon Basin, 12 locations were selected where time-dependent storm surge data for each storm are available from the ADCIRC model simulations. Our analysis was based on storm surge simulations for four hypothetical hurricanes traversing the Caernarvon Basin transect. The distances between the 12 locations were sub-sampled at equal intervals to yield 100 points (from sea to land). These points were extracted through linear interpolation of the topo-bathymetric surface to obtain wetland-water ratios, which serve as the measure of wetland continuity (*W_L_*). As *W*_L_ is measured by the ratio of wetland to water, it ranges from open water (*W_L_* = 0) to solid marsh (*W_L_* = 1). The 100 extracted points were also used to determine the spatial distribution of habitat and thus wetland roughness (*W_R_*). As *W_R_* is represented by Manning’s *n*, a measure of the bottom friction caused by the presence of wetland vegetation, it ranges from no vegetation (*W_R_* = 0.02) to high dense vegetation (*W_R_* = 0.045). **Figures 1A and 1B** display the transect location and the original gridded data used to obtain *W_L_* and *W_R_*, respectively.

Surge attenuation was then defined as the maximum reduction in storm surge per unit distance along each of the eleven transect segments defined by the 12 locations. The average length of each transect segment is approximately 6,000 m. We aggregated our extrapolated transect data on wetland continuity (*W_L_*) and wetland roughness (*W_R_*) to obtain an average value of *W_L_* and *W_R_* for each of the 11 segments. With each segment now having a unique wetland continuity and wetland roughness characteristic, it is possible to determine how varying wetland water-ratio and roughness attenuates storm surge as it traverses each transect segment.

We estimate the impact of wetland continuity and vegetation on the maximum surge for the four storms we analyzed as they traverse eleven segments of the transect (**Tables S1 and S2**). These estimated coefficients are then used to calculate the percentage change effects, or elasticities, of an increase in *W_L_* and *W_R_* per segment on the mean maximum surge level across all storms and segments (approximately 2.3 meters).

The simulations indicate that storm surges associated with these four hurricanes would impact residential property in fifteen southeastern Louisiana parishes, including the major parishes of New Orleans. The fifteen parishes are Assumption, Iberville, Jefferson, Lafourche, Livingston, Orleans, Plaquemines, St. Bernard, St. Charles, St. James, St. John the Baptist, St. Martin, St. Tammany, Tangipahoa and Terrebonne. The property damage analysis we conducted uses USACE data [27] from 312 potentially affected sub-planning units (SPUs) across these parishes, which average approximately 1,780 households per SPU with a mean residential property value of $170,701. The 312 selected SPUs were those likely impacted by a storm surge from one or more of the modeled storms.

We employ the expected damage function approach [12] to determine the marginal value of wetland continuity and vegetation in terms of reducing storm damage to residential property in the 312 potentially affected SPUs of southeastern Louisiana, including greater New Orleans. As hurricanes and storm surges are difficult to predict, the severity of economic damages inflicted may vary considerably from event to event [12], [23]–[26], [29]–[31]. We estimate the marginal value of a change in wetland-water ratio and vegetation roughness as the product of the expected marginal damage to residential property from a storm surge flood event (**Table S3**), the annual expected number of surge-flood events, and the marginal effect of wetland on the surge level estimated from our transect analysis (**Tables S1**). By combining the marginal effect on storm surge property damages with the derived probability density function for storm pressure differential (a measure of storm strength), we were able to determine the impacts of changes in wetland continuity and vegetation roughness along the storm transect on the expected marginal damage from a surge flood event in our study area (**Table 1**).

**Table 1:** Estimated storm surge impacts and marginal values of changes in wetland continuity (W_L_) and roughness (W_R_).

Estimated wetland impacts on attenuating maximumstorm surge levels (S)		Estimated marginal values of wetlands in terms of avoiding damages to residential property	
	Change in storm surge		Marginal value
1% change in W_L_ per segment	−8.4% to −11.2%	0.1 increase in W_L_ per m	$99.29 to $132.87
1% change in W_R_ per segment	−15.4% to −28.1%	0.001 increase in W_R_ per m	$23.72 to $43.24
9.4 to 12.6 km change in W_A_	−1 m	0.1 increase in W_L_ per segment	$591,886 to $792,082
		0.001 increase in W_R_ per segment	$141,399 to $257,762

W_L_ is represented by the wetland/water ratio ranging from open water (W_L_ = 0) to solid marsh (W_L_ = 1).

W_R_ is represented by Manning’s n for bottom friction caused by degree of wetland vegetation ranging from no vegetation (W_R_ = 0.02) to high density vegetation (W_R_ = 0.045).

Mean maximum surge level (S) is 2.302 m.

Mean wetland/water ratio (W_L_) is 0.408.

Mean Manning’s n (W_R_) is 0.032.

Mean transect segment length (x) is 5,961 m.

Based on Tables S1–S3.

Our model assumes that the uncertainties inherent in storm surge flood events and the resulting expected damages to property can be depicted as a Poisson process. This assumption implies that the number of potentially damaging flood events is drawn from a Poisson distribution [12], [31], [32]. Using data from the SURGEDAT database (1955–2009), we fit a Poisson distribution to the annual number of storms affecting Louisiana for which the estimated surge exceeded 0.3 m above sea level, which is the minimum surge height recorded in SURGEDAT. We found that the expected annual number of storm-surge events is 0.836 and that the Poisson distribution provides a robust fit to the observed data. This suggests that our proposed model is applicable to the modeling of storm surge events that strike the southeast Louisiana coast.

Estimating the expected marginal effect of storm surge on residential property damages requires constructing a flood damage function. Using USACE [27] flood-damage estimates at 0.3 m increments for the 312 SPUs potentially affected by a storm surge moving along the transect used for the wetland-surge level analysis (**Figure 1A and 1B**), we fit a non-linear, S-shaped curve [24]. We obtain the expected marginal effect of storm surge damages using parameter values for a Gumbel distribution of tropical storm pressure differentials [33]. From the estimated parameters obtained from the non-linear regression and the derived probability density function for storm pressure differential, we were able to determine the expected marginal damage from a surge flood event to our study area.

## Results

**Figure 2:**
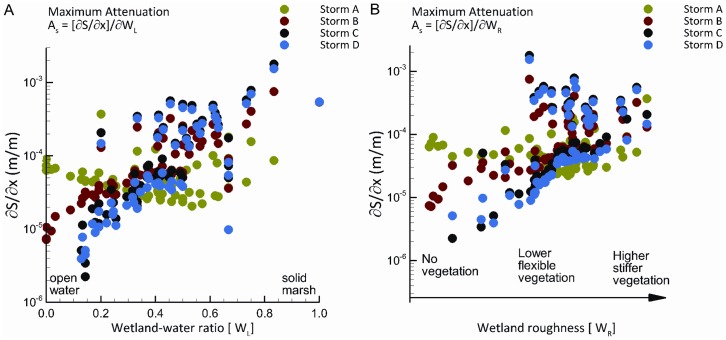
Attenuation (A_S_) of storm surge (S) as a function of wetland continuity (W_L_) and roughness (W_R_) along a storm track segment of distance (x) in m for four hurricanes in the Caernarvon Basin of southeast Louisiana. **Panel A** shows maximum attenuation as influenced by wetland continuity, 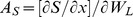, where W_L_ is represented by the wetland/water ratio ranging from open water (W_L_ = 0) to solid marsh (W_L_ = 1). **Panel B** shows maximum attenuation as influenced by wetland roughness, 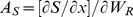, where W_R_ is represented by Manning’s n for bottom friction caused by degrees of wetland vegetation ranging from no vegetation (W_R_ = 0.02) to high dense vegetation (W_R_ = 0.045). Storm A = Central pressure of 96 kPa, radius to maximum winds (R_max_) of 67 km, forward speed of 20.5 km/hr. Storm B = Central pressure of 93 kPa, radius to maximum winds (R_max_) of 47 km, forward speed of 20.5 km/hr. Storm C = Central pressure of 96 kPa, radius to maximum winds (R_max_) of 46 km, forward speed of 20.5 km/hr. Storm D = Central pressure of 93 kPa, radius to maximum winds (R_max_) of 33 km, forward speed of 11.1 km/hr.

**Figure 2** depicts how the surge attenuation function for the four storms we analyzed is affected by both the presence of wetlands (*W_L_*) and their roughness (*W_R_*). The maximum surge attenuation associated with each storm is clearly increasing as the wetland-water ratio progresses from 0 (open water) to 1 (solid marsh) and as roughness imposed by vegetation increases from 0.02 (no vegetation) to 0.045 (high and stiff vegetation).

Our estimated elasticities, or percentage change effects, of the impact of wetland continuity and roughness on the maximum surge for each of the four storms as they traverse the eleven segments are indicated in **Table 1**. A 1% increase in the wetland-water ratio along each segment will reduce storm surge by 8.4% to 11.2%. A 1% increase in wetland roughness caused by wetland vegetation will decrease storm surge by 15.4% to 28.1%. These estimates suggest that storm surge will be reduced by 1 m per 9.4 to 12.6 km of additional wetlands along the transect we analyze.

**Table 1** also shows our corresponding estimates of the marginal value of an increase in wetland continuity and vegetation roughness in terms of reducing residential property damage from storm surge floods. A 0.1 increase in the wetland-water ratio per m along the transect (*W_L_*) will reduce flood damages by $99 to $133 for the average SPU, and a 0.001 increase in bottom friction through changes in wetland vegetation (*W_R_*) will reduce damages by $24 to $43. If such increases in wetland continuity and roughness can be extended across the landscape then damages can be further reduced. For example, an equivalent marginal increase in wetland continuity over approximately 6 km (the average length of one of our transect segments) would lower residential property flood damages by $592,000 to $792,100 for the average SPU, whereas the marginal increase in bottom friction over 6 km would reduce flood damages by $141,000 to $258,000 for the average SPU (see **Table 1**). Given the mean residential property value of $170,701 per SPU, these latter values of a marginal change in wetland continuity and vegetation roughness are equivalent to saving 3 to 5 and 1 to 2 properties per storm, respectively.

## Discussion

Without wetland restoration, Louisiana will likely lose another 4,548 km^2^ of wetland and other coastal land over the next 50 years [4]. Yet, halting wetland loss and investing in restoring wetlands is expensive. Recent estimates show that even an investment of $25 billion over 50 years still results in a loss of 585 km^2^ of wetlands [4]. Our findings, albeit for a single sea to land hurricane transect in southeastern Louisiana and a limited number of storms, show that such wetland investments could reduce the future vulnerability of the coast to periodic hurricane storm surges and decrease the risk of substantial flood damages to residential property.

Coastal wetlands globally are considered important for a wide range of ecosystem services, such as providing habitats in support of fish and other wildlife, recreation, carbon sequestration, water purification and controlling erosion [6]–[10]. But the value of temperate wetlands in protecting coastal property from storm surges may prove to be the most significant benefit, as our case study of southeastern Louisiana shows. Thus, our study confirms what many other global studies are increasingly finding, which is that the presence of wetlands can substantially reduce flooding damages from coastal storms [11]–[17].

This paper also develops a novel methodology for incorporating the influence of wetland characteristics on surge attenuation into modeling and estimating the economic value of wetlands in reducing expected property damages from hurricane storm surge. We also show that a Poisson process can be extended beyond a simply count-data approach to the number of storms to determining explicitly the distribution of expected coastal flood damages related to the distribution of storm events. This approach could be easily tested, adapted and extended to include additional sea-to-coast transects, either in southern Louisiana or other coastal areas of the Gulf of Mexico. It would also be relevant for valuing the storm protection services of other estuarine and coastal habitats, such as mangroves, sea grass beds, coral reefs and sand dunes. As better information is gained from hurricane and other tropical storm surge models, it would also be possible to improve on estimations of the distribution of expected coastal flood damages related to the distribution of storm events. Such improvements in the methodology developed here could enhance greatly future studies of the economic value of coastal wetlands in protecting property and lives.

## Supporting Information

Figure S1 **Map of the study area in southeast Louisiana, showing the location of the transect analyzed, the size and shape of the affected coastal sub-planning units (SPUs), and the locations of available time-dependent data.**
(TIFF)

Table S1 **Linear fixed effects regression of storm surge levels (S) per segment as a function of wetland continuity (** ***W_L_*** **) and roughness (** ***W_R_*** **).**
(DOC)

Table S2 **Linear fixed effects regression of storm surge levels (S) per segment as a function of wetland continuity (** ***W_L_*** **) and roughness (** ***W_R_*** **), with tests and corrections for groupwise heteroskedasticity.**
(DOC)

Table S3 **Nonlinear regression (Gauss-Newton regression) results for the residential property damage function.**
(DOC)

## References

[1] Koch EW, Barbier EB, Silliman BR, Reed DJ, Perillo GME, et al (2009) Non-linearity in ecosystem services: temporal and spatial variability in coastal protection. *Frontiers in Ecology & the Environment* 7: 29–37.

[2] Gedan KB, Kirwan MJ, Wolanski E, Barbier EB, Silliman BR (2011) The present and future role of coastal vegetation in protecting shorelines: answering recent challenges to the paradigm. *Climatic Change* 106: 7–29.

[3] Shephard CC, Crain CM, Beck MW (2012) The protective role of coastal marshes: a systematic review and meta-analysis. PLoS ONE 6: e27374. 10.1371/journal.pone.0027374 PMC3223169 22132099

[4] Coastal Protection & Restoration Authority of Louisiana (2012) *Louisiana’s Comprehensive Master Plan for a Sustainable Coast*. Office of Coastal Protection and Restoration, Baton Rouge, LA.

[5] Day JW Jr. Boesch DF, Clairain EJ, Kemp GP, Laska SB, et al (2007) Restoration of the Mississippi Delta: Lessons from Hurricanes Katrina and Rita. *Science* 315: 1679–1684. 17379799 10.1126/science.1137030

[6] Gulf Coast Ecosystem Recovery Task Force (2011) *Gulf of Mexico Regional Ecosystem Restoration Strategy.* Gulf Coast Ecosystem Recovery Task Force, Washington, D.C., December 2011.

[7] Twilley RR (2007) *Coastal Wetlands & Global Climate Change: Gulf Coast Wetland Sustainability in a Changing Climate.* Pew Center on Global Climate, Arlington, VA.

[8] Erwin KL (2009) Wetlands and global climate change: the role of wetland restoration in a changing world. *Wetlands Ecology & Management* 17: 71–84.

[9] Airoldi L, Beck MW (2007) Loss, status and trends for coastal marine habitats of Europe. *Oceanography and Marine Biology: An Annual Review* 45: 345–405.

[10] Valiela I, Kinney E, Culbert J, Peacock E, Smith S (2009) Global losses of mangroves and salt marshes. Chapter 4 in Duarte, C.M., ed. *Global Loss of Coastal Habitats: Rates, Causes and Consequences.* Fundacíon BBVA, Bilbao, Spain, 109–142.

[11] Badola R, Hussain SH (2005) Valuing ecosystem functions: an empirical study on storm protection function of Bhitarkanika mangrove ecosystem, India. *Environmental Conservation* 32: 1–8.

[12] Barbier EB (2007) Valuing ecosystem services as productive inputs. *Economic Policy* 22: 177–229.

[13] Barbier EB, Koch EW, Silliman BR, Hacker SD, Wolanski E, et al (2008) Coastal ecosystem-based management with nonlinear ecological functions and values *Science* . 321: 319–323. 10.1126/science.1150349 18202288

[14] Laso Bayas JC, Marohn C, Dercon G, Dewi S, Piepho HP, et al (2011) Influence of coastal vegetation on the 2004 tsunami wave impact in west Aceh. *Proceedings of the National Academy of Sciences* 108: 18612–18617. 10.1073/pnas.1013516108 PMC3219142 22065751

[15] Das S, Vincent JR (2009) Mangroves protected villages and reduced death toll during Indian super cyclone. *Proceedings of the National Academy of Sciences* 106: 7357–7360. 10.1073/pnas.0810440106 PMC2678660 19380735

[16] Farber S (1987) The value of coastal wetlands for protection of property against hurricane damage. *Journal of Environmental Economics and Management* 14: 143–151.

[17] Costanza R. Pérez-Maqueo O, Martinez ML, Sutton P, Anderson SJ, et al (2008) The value of coastal wetlands for hurricane protection *Ambio* . 37: 241–248. 10.1579/0044-7447(2008)37[241:tvocwf]2.0.co;2 18686502

[18] Loder NM, Irish JL, Cialone MA, Wamsley TV (2009) Sensitivity of hurricane surge to morphological parameters of coastal wetlands. *Estuarine, Coastal and Shelf Science* 84: 625–636.

[19] Wamsley TV, Cialone MA, Smith JM, Atkinson JH, Rosati JD (2010) The potential of wetlands in reducing storm surge *Ocean Engineering* . 37: 59–68.

[20] Resio DT, Westerink JJ (2008) Hurricanes and the physics of surges. *Physics Today* 61: 33–38.

[21] Westerink JJ, Luetich RA, Feyen JC, Atkinson JH, Dawson C, et al (2008) A basin to channel-scale unstructured grid hurricane storm surge model applied to southern Louisiana. *Monthly Weather Review* 136: 833–864.

[22] Westerink JJ, Dietrich J, Bunya S, Westerink H, Tanaka S, et al. (2007) “Flood Insurance Study: Southeastern Parishes, Louisiana - Intermediate Submission 2.: Offshore Water Levels and Waves.” U.S. Army Corps of Engineers, New Orleans.

[23] Merz B, Kreibich H, Schwarze R, Thieken A (2010) Review article: assessment of economic flood damage. *Natural Hazards and Earth System Science* 10: 1697–1724.

[24] Burrus RT, Dumas CF, Graham JE (2001) The cost of coastal storm surge damage reduction. *Cost Engineering* 43: 38–44.

[25] Dutta D, Herath S, Musiake K (2003) A mathematical model for flood loss estimation. Journal of Hydrology 277: 24–49.

[26] Pistrika AK, Jonkman SN (2009) Damage to residential buildings due to flooding of New Orleans after hurricane Katrina. *Natural Hazards* 54: 413–434.

[27] US Army Corps of Engineers (USACE) (2006) *Catalog of Residential Depth-Damage Functions Used by the Army Corps of Engineers in Flood Damage Estimation.* USACE, Washington, DC.

[28] Vigdor J (2008) The economic aftermath of Hurricane Katrina, *Journal of Economic Perspectives* . 22: 135–154.

[29] Merz B, Kreibich H, Thieken A, Schmidtke R (2004) Estimation uncertainty of direct monetary flood damage to buildings *Natural Hazards and Earth System Science* . 4: 153–163.

[30] Pielke RA Jr, Gratz J, Landsea CW, Collins D, Saunders MA, et al (2008) Normalized hurricane damage in the United States: 1900–2005. *Natural Hazards Review* 9: 29–42.

[31] Katz RW (2002) Stochastic modeling of hurricane damage *Journal of Applied Meteorology* . 41: 754–763.

[32] Bengtsson A, Nilsson C (2007) Extreme value modeling of storm damage in Swedish forests *Natural Hazards and Earth System Science* . 4: 515–521.

[33] Resio DT (2007) White paper on estimating hurricane inundation probabilities. Engineer Research and Development Center (ERDC), US Army Corps of Engineers, Vicksburg, MI.

