# Supplementary figures and images for: The Value of Wetlands in Protecting Southeast Louisiana from Hurricane Storm Surges
Source: PLoS One. 2013 Mar 11;8(3):e58715. doi: 10.1371/journal.pone.0058715 (PMC3594144; doi:10.1371/journal.pone.0058715)

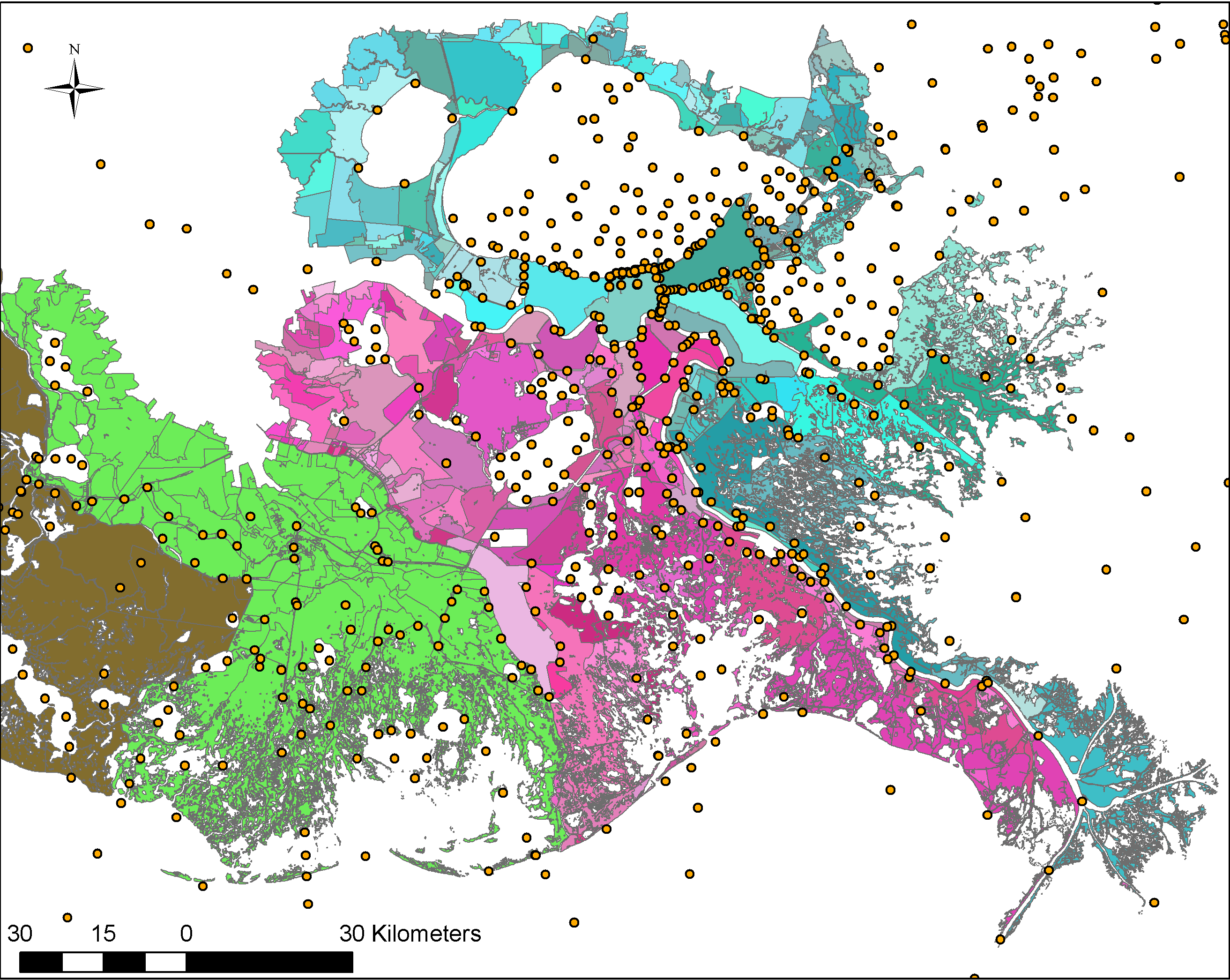

Supplement: Figure S1 — Map of the study area in southeast Louisiana, showing the location of the transect analyzed, the size and shape of the affected coastal sub-planning units (SPUs), and the locations of available time-dependent data. (TIFF) [file pone.0058715.s001.tiff]
