# Supplementary material for: The Value of Wetlands in Protecting Southeast Louisiana from Hurricane Storm Surges
Source: PLoS One. 2013 Mar 11;8(3):e58715. doi: 10.1371/journal.pone.0058715 (PMC3594144; doi:10.1371/journal.pone.0058715)
Supplement: Table S2 — Linear fixed effects regression of storm surge levels (S) per segment as a function of wetland continuity ( WL ) and roughness ( WR ), with tests and corrections for groupwise heteroskedasticity. (DOC) [file pone.0058715.s003.doc]

**Table S2.** Linear fixed effects regression of storm surge levels (S) per segment as a function of wetland continuity (*W_L_*) and roughness (*W_R_*), with tests and corrections for groupwise heteroskedasticity

| **VARIABLE** | **Fixed Effects** | **Corrected Fixed Effects (FGLS)** |
| --- | --- | --- |
| **Constant** | 2.171**  (0.138) | 2.184**  (0.126) |
| **Land-Water Ratio**  **(Wetland Continuity)** | -0.473**  (0.121) | -0.479**  (0.108) |
| **Roughness (Manning’s n)** | -11.263*  (4.511) | -11.593**  (4.009) |
| **Storm A**  **(Dummy)** | 1.127**  (0.088) | 1.127**  (0.077) |
| **Storm B**  **(Dummy)** | -0.408**  (0.088) | -0.408**  (0.089) |
| **Storm C**  **(Dummy)** | 2.000**  (0.088) | 2.000**  (0.086) |
|  | ***F=193.67*****  ***R^2^=0.962***  ***Adj R^2^= 0.957***  ***AIC = -0.190***  ***Wald = 95.218*****  ***LM = 1.397*** | ***Log L = 11.064*****  ***χ*^2^ = 1.758** |

n (number of observations) = 44

Standard errors are indicated in parentheses below each estimated coefficient.

*Significant at 95% confidence level

**Significant at 99% confidence level

Mean maximum surge level (S) is 2.302 m

Mean wetland/water ratio (W_L_) is 0.408

Mean Manning's n (W_R_) is 0.032

Mean transect segment length (x) is 5,961 m

FGLS = feasible generalized least squares
