# Supplementary material for: The Value of Wetlands in Protecting Southeast Louisiana from Hurricane Storm Surges
Source: PLoS One. 2013 Mar 11;8(3):e58715. doi: 10.1371/journal.pone.0058715 (PMC3594144; doi:10.1371/journal.pone.0058715)
Supplement: Table S3 — Nonlinear regression (Gauss-Newton regression) results for the residential property damage function. (DOC) [file pone.0058715.s004.doc]

**Table S3.** Nonlinear regression (Gauss-Newton regression) results for the residential property damage function

| **Parameter** | **Regression Estimate**  **(R^2^ = 0.98)** |
| --- | --- |
| ***α_F_*** | 0.0001**  (0.00001) |
| ***β_1F_*** | 3.671**  (0.04861) |
| ***β_2F_*** | 0.991**  (0.00017) |
| ***H_0_: β_1F_=β_2F_ =1*** | *F=7,009.8*** |
| ***H_0_: β_1F_=1*** | F=7894.92**  W=3,018** |
| ***H_0_: β_2F_=1*** | F=1,828.8**  W=2909.65** |
|  |  |

See equation (18).

Mean residential property value per SPU is $170,701

Mean level of surge is 1.55 feet (0.472 m).
